# Aging, longevity, and the role of environmental stressors: a focus on wildfire smoke and air quality

**DOI:** 10.3389/ftox.2023.1267667

**Published:** 2023-10-11

**Authors:** David Scieszka, Alicia M. Bolt, Mark A. McCormick, Jonathan L. Brigman, Matthew J. Campen

**Affiliations:** ^1^ Department of Pharmaceutical Sciences, College of Pharmacy, University of New Mexico Health Sciences Center, Albuquerque, NM, United States; ^2^ Department of Biochemistry and Molecular Biology, School of Medicine, University of New Mexico Health Sciences Center, Albuquerque, NM, United States; ^3^ Department of Neurosciences, School of Medicine, University of New Mexico Health Sciences Center, Albuquerque, NM, United States

**Keywords:** aging, environmental exposures, environmental stressors, longevity, wildfires, wildfire smoke, air quality

## Abstract

Aging is a complex biological process involving multiple interacting mechanisms and is being increasingly linked to environmental exposures such as wildfire smoke. In this review, we detail the hallmarks of aging, emphasizing the role of telomere attrition, cellular senescence, epigenetic alterations, proteostasis, genomic instability, and mitochondrial dysfunction, while also exploring integrative hallmarks - altered intercellular communication and stem cell exhaustion. Within each hallmark of aging, our review explores how environmental disasters like wildfires, and their resultant inhaled toxicants, interact with these aging mechanisms. The intersection between aging and environmental exposures, especially high-concentration insults from wildfires, remains under-studied. Preliminary evidence, from our group and others, suggests that inhaled wildfire smoke can accelerate markers of neurological aging and reduce learning capabilities. This is likely mediated by the augmentation of circulatory factors that compromise vascular and blood-brain barrier integrity, induce chronic neuroinflammation, and promote age-associated proteinopathy-related outcomes. Moreover, wildfire smoke may induce a reduced metabolic, senescent cellular phenotype. Future interventions could potentially leverage combined anti-inflammatory and NAD + boosting compounds to counter these effects. This review underscores the critical need to study the intricate interplay between environmental factors and the biological mechanisms of aging to pave the way for effective interventions.

## 1 Introduction

You are going to die (>95% confidence). Odds are strong that the cause of your death will be age-related. Ample scientific evidence suggests that contaminants in our environment, including air pollution (Pope et al., 2009), can not only contribute to the cause of death, but also as a result shorten lifespan. However, there is also common thought that environmental challenges (for instance caloric restriction and fasting) may enhance cellular and organismal efficiency to promote longevity. A growing number of scientists believe this mortal fate can be altered, and death might not be a guarantee, as certain organisms and cells have been effectively “immortalized”. “Why humans age” is a timeless question, with recorded answers dating back to Aristotle’s theory of aging. His comprehension of biology was understandably limited, and he was tethered with terminology that was more romanticized and metaphorical. Yet, he surmised that the aging process coincides with the growth and decay of lungs, the exhaustion of heat in the heart, and a general cooling as we become geriatric (Aristotle, 2023). Unfortunately, the question of “why we age” was largely disregarded by the scientific community for nearly 1,000 years thereafter.

During the Darwinian era of science, researchers believed that aging was a natural aspect of evolution, with intervention being an impossibility. Furthermore, most humans during this period of history died of infectious diseases, deeming scientific investigation in aging as unnecessary. These feelings of indifference would change in the early 1900’s after scientists realized that mortality rate of diseases increased in concert with age. Through intervention studies, the caloric restriction theory showed promise in slowing down the process of aging and extending lifespan (Holehan and Merry, 1986).

Fortunately, humans have significantly advanced our ability to both investigate biological mechanisms and alter the natural outcomes of life. In the early 1970’s and 1980’s, nematodes were employed by Dr. Michael R. Klass and others to determine genetic variations that could prolong lifespan. To date, the list of longevity-associated genes has risen to 115 in total, spanning 25 species (Yu et al., 2021; Bin-Jumah et al., 2022). Aging research sprawled outward in all directions, looking for any and every way possible to increase longevity. One group even found that the removal of gonads will increase lifespan (Hsin and Kenyon, 1999).

**FIGURE 1:**
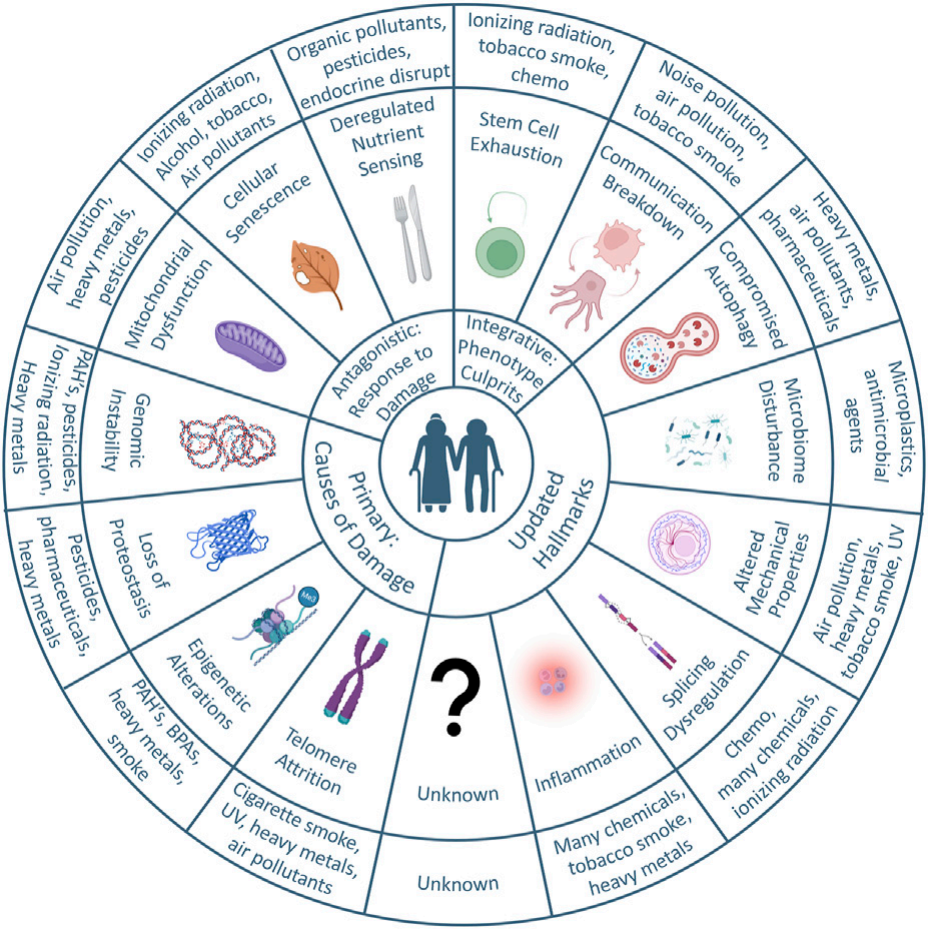
Graphical abstract: the hallmarks of aging and environmental contributors. Adapted from the original and updated hallmarks of aging, the circular plot illustrates the concepts known to contribute to aging thus far and the potential influences of environmental toxicants. The circle immediately out from the center illustrates the main categories. Primary hallmarks are the causes of damage, and include telomere attrition, epigenetic alterations, loss of proteostasis, and genomic instability. Antagonistic hallmarks are responses to damage, and include mitochondrial dysfunction, cellular senescence, and deregulated nutrient sensing. Integrative hallmarks manifest via the aging phenotype and include stem cell exhaustion and communication breakdown. The Hallmarks were updated after a 2022 Aging Research and Drug Discovery meeting. The most outer circle illustrates the environmental toxicants that are known to affect each of the primary, antagonistic, integrative, or updated hallmarks.

Some trends (thankfully) never made it to the clinic. But, through a massive undertaking, consistencies emerged, mechanisms of aging were theorized, and some have been popularized. We now understand that human longevity is modestly heritable - somewhere between 12% and 25% (Herskind et al., 1996; Kaplanis et al., 2018; Ruby et al., 2018; van den Berg et al., 2017; van den Berg et al., 2019)- with family clusters showing a further increase in longevity incidence (Pedersen et al., 2017). Enhancements for these families include insulin sensitivity (Wijsman et al., 2011) and lipid metabolism, leading to healthy lipid levels in the circulatory system (Vaarhorst et al., 2011). Additionally, some families are observed to have more robust immune systems and metabolisms, leading to extensions in longevity (Andersen et al., 2012; Ash et al., 2015). However, the question of “why humans age” still persists, with our current best guesses revolving around the López-Otín et al. (2013) publication of the 9 hallmarks of aging in 2013; updated to 15 hallmarks in 2022 (Schmauck-Medina et al., 2022) (Figure 1).

The original hallmarks fall into 3 main categories of damage-causing (primary), response to damage (antagonistic), and those that manifest via the aging phenotype (integrative). Primary hallmarks that cause damage include genomic instability, telomere attrition, epigenetic alterations, and loss of proteostasis. From antagonistic damaging stimuli, our bodies can be subjected to deregulated nutrient sensing, mitochondrial dysfunction, and cellular senescence. With accumulated damage, we manifest the aging phenotype through stem cell exhaustion and altered intercellular communication. Strangely, the hallmarks themselves are not guaranteed as “causes of aging” but could also be viewed as “the reason for mortality.” To illustrate, we will frame this argument through the lens of common model organisms and their “reason for mortality.” Typically, cancer is the leading cause of mortality across mouse strains (70%–90%) (Leibiger and Berggren, 2006; Brady and Attardi, 2010; Feng et al., 2011; Ng et al., 2015; Zhao et al., 2018b).

A meta-analysis was performed on the putative longevity interventions in mouse models, which revealed >80% of interventions are linked to cancer inhibition (Keshavarz et al., 2022). As such, those longevity interventions linked to cancer incidence prevention could be viewed as “mortality reduction” rather than “aging interventions”. Just as the cause of death in mice is typically cancer, reductions in intestinal stem cell function can be linked to the cause of death in *Drosophila melanogaster* (Rera et al., 2012). From genetic manipulation studies, several longevity genes have been targeted to reduce intestinal stem cell hyperproliferation, which resulted in an extended lifespan (Waters and Kaye, 2002; Mahesh and Kaskel, 2008; van Heemst, 2010; Xing, 2010; Vijayakumar et al., 2011; López-Otín et al., 2016; Bartke, 2019). In yeast, increases to extrachromosomal circular DNA elements (eccDNA) have been linked with mortality (Sinclair and Guarente, 1997). This was reversed through sirtuin activation, resulting in lifespan extension. For *C. elegans*, the natural cause of death is not fully understood. Studies have recently pointed toward a positive correlation between active pharyngeal pumping with longevity (de Zelicourt et al., 2016). These results were expanded by observing two types of death. Namely, an infected pharynx leading to early mortality, and pharyngeal atrophy leading to longevity (Zhu et al., 2002). Regardless, with recent advances in *Caenorhabditis elegans* lifespan study automation (Felker et al., 2020) and high throughput data analysis pipelines (Small et al., 2020), the “reason for mortality” could soon come into focus.

While a great deal of effort–and progress–has been made in recent years understanding the basic molecular and cellular pathways that promote aging, the role of environmental stressors has not yet entered into the “hallmarks of aging” lexicon. When examining aging from a bottom-up approach, it is a complicated, multidimensional issue with contributions from all levels of cellular health. To fully grasp this, one would need to understand the aging-associated input and interactions from the molecular level of each cell, build that understanding in a stepwise manner to the level of tissue organization, and deconvolute the crosstalk from an individual layer before interlayer dependencies could be understood. Adding the implications of xenobiotic agents/chemicals to this mix is an exponential increase in complexity. Fortunately, scientific advancements are not restricted to an all-or-nothing approach. From the top-down perspective, research has shown that chemicals in our environment exist that can impact each and every hallmark of aging.

For example, exposure to heavy metals can induce oxidative stress, linking exposure to the aging hallmarks of mitochondrial dysfunction and DNA damage. These same metals can directly inhibit DNA repair. Therefore, these metals can be implicated in the hallmarks of epigenetic alteration, genomic instabilty, loss of proteostasis, and compromised autophagy. There have also been direct epidemiological links between air pollution and a decrease in life expectancies. Notably, C. Adren Pope et al. and others found that a reduction in air pollution of 10 μg/m^3^ was associated with an increased mean life expectancy of 0.17 years (Pope et al., 2009; Correia et al., 2013). For context, wildfires consistently generate air pollution levels well in excess of 100 μg/m^3^ for weeks at a time. Others have sought to determine mechanistic understandings, and found DNA damage events in the lung (Kaur et al., 2021), vascular endothelial activation (Aragon et al., 2016) and neuroinflammatory effects in the brain (Haghani et al., 2020; Scieszka et al., 2022). This inexorable linkage between the environment and each hallmark of aging is explored in more detail below.

## 2 Primary hallmarks of aging: genomic instability, telomere attrition, epigenetic alterations and clocks, and loss of Proteostasis

### 2.1 Genomic instability

Genomic instability is defined as an increased susceptibility to mutational frequency and other genetic alterations during cellular division. Genomic instability is a prerequisite for many cancers, is modestly heritable, clearly vulnerable to environmental toxicants, and increases with age (Vijg and Suh, 2013; Vijg and Montagna, 2017). Regarding cancer, the systems in charge of genomic PMCRS (preventative maintenance, checks, repair, and service) have failed, causing dysregulated cellular division. The heritability of genomic instability mainly arises from conferred mutations in DNA repair enzymes themselves, or other molecules that stabilize cellular processes during division (NCI Dictionary of Cancer Terms - NCI, 2011). However, genomic instability can also arise from environmental toxicant exposures, including heavy metals like chromium, cadmium, and arsenic (Langie et al., 2015; Balali-Mood et al., 2021; Medina et al., 2022). Specifically, arsenic is known to cause epigenetic changes, disruptions to DNA base excision repair and nonhomologous end joining, increases to oxidative stress, abnormal apoptotic signaling, impaired lineage commitment of hematopoietic progenitors, and immunosuppression through autophagy alteration (Bolt et al., 2010a; Bolt et al., 2010b; Bolt et al., 2012; Bolt and Klimecki, 2012; Medina et al., 2017; Medina et al., 2020; Medina et al., 2021; Parvez et al., 2017; Zhou et al., 2020). Mechanistically, arsenic biotransformation can cause s-adenosylmethionine depletion leading to epigenetic alterations, while different forms of downstream monomethylarsonic acid and dimethylarsinic acid cause increases to ROS generation, leading to DNA damage and mitochondrial dysfunction (Minatel et al., 2018). Biotransformation products of arsenic are also known to compete with zinc finger domains in transcription factors and DNA repair enzymes, such as such as GATA-1, GATA-2, PARP-1, XPC, APE1, OGG-1, XRCC, and ERCC (Minatel et al., 2018; Medina et al., 2022). The simultaneous damage of DNA and inhibition of repair mechanisms can lead to pathogenic outcomes like cancer, or cell-cycle inhibiting results that lead to senescence or apoptosis, among others.

Somewhat paradoxically, genomic instability can also confer cellular stability. Of note, extrachromosomal circular DNA (eccDNA) is a type of circular, double-stranded DNA element within the nucleus. Studies have found that these exist in healthy (Møller et al., 2018), cancerous (Fan et al., 2011), and aged cells (Hull et al., 2019), with many concluding that these elements drive tumor formation (Turner et al., 2017) due to enhanced chromatin accessibility (Wu et al., 2019). However, Shoura et al. (2017) found eccDNA in worm germ line cells, which begs the question of heritability. In humans, some eccDNAs originate from telomeres (t-circles) and from centromeres (Zuo et al., 2022). The consistency between these two are that they both consist of tandem repeats, but the functionality of tandem repeats remains unknown, with recent work suggesting translational possibilities (Al-Turki and Griffith, 2023). In cell culture, t-circles were required for healthy telomere maintenance (Neumann et al., 2013), and excreted eccDNAs have been found *in vitro*. Here, they are reported as messengers that mousee and human cells recognize (Wang et al., 2021b). Based on the accepted eccDNA formation mechanisms, one could speculate that lucky cells might acquire gain-of-function eccDNAs that confer longevity. This is based on the recently found gain of function enhancement to transcription factor repair fidelity (Xu et al., 2021). Alternative to genomic *instability*, the heritability of genomic *stability* is woefully understudied. In fact, the majority of studies were only able to identify a small number of genes with variations. Namely, variants for *APOE* and *FOXO3A* (Nygaard et al., 2014; Flachsbart et al., 2017) that could confer longevity in individuals across generations. The variants in *APOE* and *FOXO3A* genes have been shown to play a role in regulating DNA repair and oxidative stress response, respectively. However, the complexity of the human genome and the interactions between different genetic and environmental factors make it challenging to fully understand the heritability of genomic stability.

### 2.2 Telomere attrition

Perhaps one of the best-studied hallmarks of aging, telomere attrition has been linked to somatic aging (Vaiserman and Krasnienkov, 2021) and artificially extending telomeres has been found to reverse signs of aging in rodents (Jaskelioff et al., 2011). However, the predictive framework and ability to reverse telomere-related aging effects have seen mixed results. Succinctly put, the replicative ability of cells has a limit, and this limit has been eponymously named the Hayflick Limit (Hayflick and Moorhead, 1961) *after a series of experiments performed by Leonard Hayflick in 1961.* Embryonic stem cells activate the gene for telomerase reverse transcriptase, an enzyme that extends the end-caps of chromosomes, called telomeres. However, most adult stem cells lack telomerase expression.

After each cellular replication, telomeric DNA shortens by 50–200 base pairs due to incomplete lagging strand synthesis (Srinivas et al., 2020). DNA polymerases are unable to fully replicate the 3′ DNA sequence, leading to “the end-replication problem” (Watson, 1972; Olovnikov, 1973). Telomere length is highly heterogeneous between tissue types. It has also been shown that telomeres are highly susceptible to oxidative damage (Oikawa and Kawanishi, 1999), meaning lifestyle choices and environmental exposures can directly affect the length of cellular telomeres. Regardless, successive cellular divisions by stem cells will eventually lead to full telomere attrition, resulting in cell cycle arrest. As such, the length of telomeres was thought to be associated with the biological somatic age of humans, and has been proposed as a key driver of aging (von Zglinicki et al., 2021). These studies reached a fever pitch when the CEO of BioViva, Elizabeth Parrish, traveled to Columbia for a gene therapy injection designed to activate her cells’ telomerase gene. BioViva claimed that her immune cells showed a reduction in “telomeric age” of 20 years in some cases. Unfortunately, this study lacked US government approval, had an *n* = 1, and was not widely promoted within the academic community.

Regarding environmental exposures, air pollution has been shown to shorten telomere length in several cell types, including leukocytes, placenta, and lung epithelial cells (Zhao et al., 2018a; Chang-Chien et al., 2021), which directly correlates with accelerated aging (Pieters et al., 2016), and cognitive decline in the elderly (Colicino et al., 2017). Alarmingly, prenatal exposure to air pollution resulted in shorter leukocyte telomere length at birth (Lee et al., 2020; Durham et al., 2022). One follow-up study showed these prenatally exposed children had a significant increase in repeated wheeze tests 4 years later. How the lungs of unborn children are subject to leukocyte telomere effects was not discussed. However, these results are a stark reminder of the interconnectedness of organ systems and the continued linkage of environmental exposures with aging.

### 2.3 Epigenetic alterations and clocks

**FIGURE 2:**
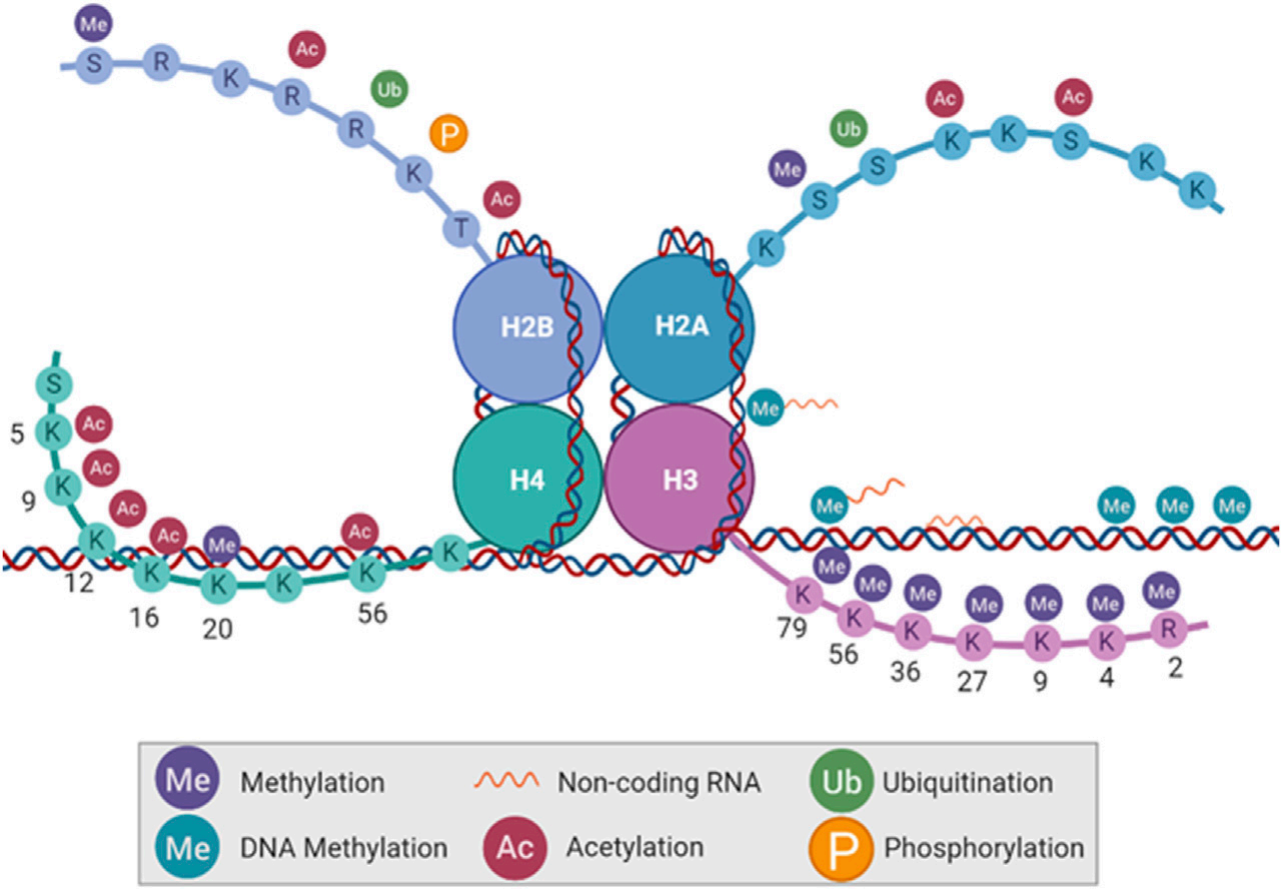
Types of epigenetic modifications. DNA base pairs and histone tail amino acid residues can be altered without changing the DNA code itself. Histone tails can be methylated, acetylated, ubiquitinated, and phosphorylated. DNA can be methylated as well, with the absence or addition of non-coding RNA.

By root definition, an epigenetic alteration is an alteration that is above the genetic code. These alterations do not change the genetic code itself but alter the expression of genes through modifications to bases in heterochromatin and euchromatin, leading to increased or decreased accessibility. Histone tails can be rapidly modified before, during, and after the cell cycle via methylation, acetylation, ubiquitination, and phosphorylation on different amino acid residues. Conversely, the DNA has a relatively stable presence of methyl groups, noncoding RNAs (ncRNA) bound to methyl groups, or ncRNAs bound to the DNA sequence itself (Figure 2). Histones can also be methylated, but it is much less common. All of these alterations serve to change the effects of transcription, DNA accessibility, and mitotic bookmarks.

For a brief history, evidence of transcriptional changes based on epigenetic alterations began in the 1960’s through direct DNA and RNA comparisons at different timepoints during whale pregnancy (Berdyshev et al., 1967). However, the ability to directly measure the methylation status of particular DNA loci was not popularized until the 1980s through the use of methylation-sensitive restriction enzymes. These loci are often referred to as CpG islands, which are defined as clusters of Cytosine-phosphate-Guanine dinucleotides and are the target of DNA methyltransferases.

Due to the DNA methylome being relatively stable, methyl group additions play a critical role in gene silencing. These can be additions in gene promoter regions, which affect transcription factor binding, or alterations to histones and their tails, resulting in heterochromatin (Esteller, 2008). Regarding histone tails, >60 post-translational modifications have been identified (Sterner and Berger, 2000; Cuthbert et al., 2004; Nowak and Corces, 2004; Nathan et al., 2006; Nelson et al., 2006; Shilatifard, 2006; Berger, 2007; Goldberg et al., 2007). Regarding ncRNAs, they can act independently or in addition to other epigenetic modifications to silence genes or maintain gene silencing. For an in-depth review of these topics, there are a plethora of publications available (Sterner and Berger, 2000; Cuthbert et al., 2004; Nowak and Corces, 2004; Nelson et al., 2006; Shilatifard, 2006; Trojer and Reinberg, 2006; Berger, 2007; Goldberg et al., 2007; Jones and Baylin, 2007; Ruthenburg et al., 2007; Gal-Yam et al., 2008).

Regarding environmental exposures, air pollution has been shown to modulate the epigenetic landscape differentially throughout lifespan based on the onset and duration of exposure (Ferrari et al., 2019). Broadly, prenatal exposures have been linked with global alterations, with specific focus on long interspaced nuclear elements 1 (LINE-1) and its function of telomere reprogramming during embryo development (Wang et al., 2021a; Kohlrausch et al., 2022); childhood alterations are varied and are increased with asthma; and adult alterations are fewer than those seen during childhood but are greatly increased by disease states. For an overview adapted from Ferrari et al. (2019), see Table 1. With respect to functional outcomes, air pollution-induced epigenetic changes have been associated with lung cancer (Li et al., 2017), COPD (Leclercq et al., 2017), cardiovascular disease (Chen et al., 2018), atherosclerosis (Chi et al., 2016), Alzheimer’s disease (Calderón-Garcidueñas et al., 2020), accelerated biological aging (Ward-Caviness et al., 2016) and many others (Wilhelm-Benartzi et al., 2011; Janssen et al., 2013; Liu et al., 2015; Breton et al., 2016; Byun et al., 2016; Motta et al., 2016; Ding et al., 2017; Solaimani et al., 2017; Li et al., 2018; Maghbooli et al., 2018; Miller et al., 2018; Rodosthenous et al., 2018; Callahan et al., 2019; Goodson et al., 2019; Gruzieva et al., 2019; Cantone et al., 2020; Eze et al., 2020; Ma et al., 2020; Tsamou et al., 2020; Liang et al., 2021; Mukherjee et al., 2021).

**TABLE 1:** Effects on methylation changes from pollution exposure at different stages of lifespan. Adapted from Ferrari et al. (2019).

Life stage	↑/↓*	Genes	Type	Tissue	Ref.
Preconception	↑	Global	Mouse	Sperm	Yauk et al. (2008)
Pregnancy, first trimester	↑	LINE-1	Human	Blood spots	Breton et al. (2016)
Pregnancy, first trimester	↓	Global	Human	Placenta	Janssen et al. (2013)
Pregnancy, first trimester	↓	LINE-1	Human	Placenta	Cai et al. (2017)
Pregnancy	↓	LINE-1	Human	Placenta	Kingsley et al. (2016)
Pregnancy	↑↓	7 CpG sites (i.e., three located near PTPRN2, TMEM125, and VPS4A genes, the other 4 sites mapped to non-genic regions)	Human	Placenta	Kingsley et al. (2016)
Pregnancy, first and second trimester	↑	HSD11B2	Human	Placenta	Oakley and Cidlowski (2013)
Pregnancy, second trimester	↑	SOD2	Human	Cord blood and maternal blood	Zhou et al. (2020)
Pregnancy	↑↓	APEX1, OGG1, ERCC4, TP53 DAPK1	Human	Placenta	Neven et al. (2018)
Pregnancy, third trimester	↑	NPAS2, CRY1, PER2, PER3	Human	Placenta	Nawrot et al. (2018)
Pregnancy, first trimester	↑	D-loop, MT-RNR1	Human	Placenta	Janssen et al. (2013)
Childhood, asthma	↓	Immune genes (e.g., IL13 and RUNX3)	Human	Blood	Yang et al. (2016)
Childhood, asthma	↑	FOXP3	Human	Blood	Prunicki et al. (2018)
Childhood	↓	IL-4, IFN-γ	Human	Blood	Jung et al. (2017)
Childhood	↓	TET1	Human	Nasal airway cells	Somineni et al. (2016)
Childhood	↑	FAM13A, NOTCH4	Human	Blood	Gruzieva et al. (2019)
Adult age, healthy	↑↓	MATN4, ARPP21, CFTR	Human	Blood	Gondalia et al. (2019)
Adult age, obese	↓	CD14, TLR4	Human	Blood	Cantone et al. (2020)
Adult age, occupational exposure	↓	NOS3, EDN1	Human	Blood	Tarantini et al. (2013)
Adult age, CVD	↑↓	cg20455854, cg07855639, cg07598385, cg17360854, cg23599683	Human	Blood	Chi et al. (2016)
Adult age, CVD	↓	Global	Human	Blood	Plusquin et al. (2017)
Adult age, CVD	↓	Alu, TLR4	Human, crossover	Blood	Bellavia et al. (2013)
Adult age, CVD	↑	IFN-γ	Human, crossover	Blood	Tobaldini et al. (2018)
Adult age, CVD	↑↓	Loci related to insulin resistance, glucose and lipid metabolism, inflammation, oxidative stress, platelet activation, and cell survival and apoptosis	Human, crossover	Blood	Li et al. (2018)
Adult age, CVD	↑↓	Loci related to apoptosis, cell death and metabolic pathways, or associated with ion binding and shuttling	*In vitro*	Human cardiomyocytes AC16	Yang et al. (2017)
Adult age, respiratory disease	↑↓	2,827 CpG sites (genes involved in inflammation and oxidative stress response), repetitive elements, and microRNA	Human, crossover	Blood	Jiang et al. (2013)
Adult age, respiratory disease	↑↓	12 differentially methylated probes and 27 differentially methylated regions	Human	Blood	Lee et al. (2020)
Adult age, cancer	↑	P16^INK4A^	*In vitro*	*Ex vivo* lymphocytes	Fougere et al. (2018)
Adult age, cancer	↑	P16^INK4A^	*In vitro*	Primary human bronchial epithelial cells	Leclercq et al. (2017)
Adult age, cancer	↑↓	66 genes	*In vitro*	BEAS-2B cells	Hesselbach et al. (2017)
Adult age, cancer	↑↓	P16^INK4A^, APC, LINE-1, NOS2	Mouse	Blood	Ding et al. (2017)
Adult age, cancer, DOHaD	↑↓	*SYK*, *CCND2*	Human	Blood	Callahan et al. (2019)
Adult age	↑	MT-TF, MT-RNR1	Human	Blood	Byun et al. (2013)
Adult age	↓	D-loop	Human	Blood	Byun et al. (2016)
Elderly	↑↓	Genes involved in tumor development, gene regulation, inflammatory stimuli, pulmonary disorders, and glucose metabolism	Human	Blood	Panni et al. (2016)
Elderly	↑↓	LINE-1, Alu, IL-6	Human	Blood	Bind et al. (2012)
Elderly	↓	iNOS	Human	Blood	Madrigano et al. (2012)

**FIGURE 3:**
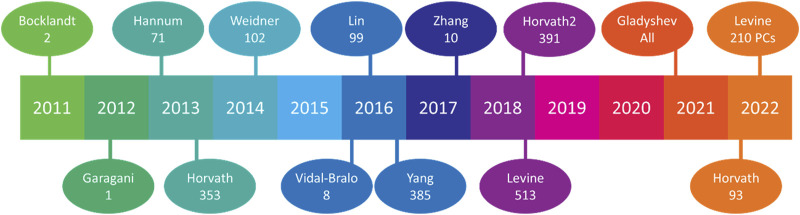
Aging clocks released by year. Since 2011, 14 aging clocks have been widely publicized, with their respective methylation sites beneath the lead author’s name. Horvath’s 2013 release featured the first multi-tissue clock, Vidal-Bralo’s utilized a validation cohort, Yang’s sought to clock CD4 monocytes, Zhang attempted to predict ACM (Arrhythmogenic cardiomyopathy), Gladyshev used all methylation sites found from scRNA-seq analysis, and Levine’s 2022 release utilized different principal components (PCs) from methylation sites rather than methylation sites themselves.

Some of the contemporary excitement in epigenetics involves epigenetic clocks. These clocks measure DNA methylation sites to correlate a “biological” or “phenotypic” age to the “chronological” age and are prized for their ability to measure longevity rejuvenation therapeutic effectiveness. Although the field has developed many aging clocks (Figure 3), their nuances have increased, and the causal nature of these methylation sites is still incompletely understood. Meaning, they originally sought to measure the biological age and correlate it to chronological age to determine aging trajectories. However, people have developed clocks for children, clocks for women/men exclusively, clocks for cell culture, clocks to measure physical fitness, clocks for blood, clocks for skin, and many have repurposed clocks to measure exposome-based age accelerations. These clocks will give you a snapshot of the current phenotypic age. But they will not tell you whether these methylation sites are causing a change in phenotypic age relative to chronological, or whether these sites are the result of the change. Unsurprisingly, air pollution particulate matter content, size, and location of population (high or lower pollution) have altered clock results. For example, one group sampled non-Hispanic white women aged 35–47 in the United States (*n* = 2,764) and found age acceleration associated with particulate matter <10 microns in diameter (PM_10_), with no correlation between age and NO_2_, and heterogeneity per cluster when measuring PM_2.5_ correlations (White et al., 2019b). Contrarily, the same group separated the US by multi-state regions and subdivided further by county level. They found PM_2.5_ to be associated with epigenetic age, while NO_2_ was inversely related, and PM_10_ had no correlation (White et al., 2019a). These studies offer intriguing findings, but also indicate the novelty of air pollution within the aging research space. Other groups have investigated the interaction of differential effects of environmental toxicants with epigenetic outcomes, such as microplastics, cadmium, and arsenic (Meakin et al., 2019; Zhang et al., 2020).

### 2.4 Loss of proteostasis

This hallmark describes the breakdown of protein building machinery, an accumulation of misfolded proteins, and the link to age-related diseases, like Alzheimer’s disease and related dementias (ADRD). The loss protein homeostasis has subcategories which can be divided into dysregulation/breakdown of ribosomal pathways, chaperones, and protein degradation.

Regarding ribosomes, extrachromosomal ribosomal circular DNA (ecrDNA) appears to be a key factor in the aging process (Hull and Houseley, 2020). Within yeast, ecrDNA molecules were discovered by Guarente’s group at MIT in 1997 (Sinclair and Guarente, 1997). These consisted of ribosomal DNA repeats, and titration of ecrDNA abundance was further proposed to limit the lifespan of yeast mothers. This belief is widely regarded in the literature as correct, however many studies have found no correlation with ecrDNA abundance and lifespan (Heo et al., 1999; Kim et al., 1999; Mays Hoopes et al., 2002; Merker and Klein, 2002; Borghouts et al., 2004; Kaeberlein et al., 2005; Ganley et al., 2009; Kwan et al., 2013). A second theory regarding ecrDNA maintenance has been proposed as the main driver of this yeast aging phenotype (Sinclair and Guarente, 1997). Ribosomal DNA is repeated a large number of times in most eukaryotes, with rDNA representing two of the largest repeats within humans (Warmerdam and Wolthuis, 2019). However, the nature of repeated DNA segments makes their replication more prone to errors during recombination events (Carvalho and Lupski, 2016; Warmerdam and Wolthuis, 2019), and these repeats have been shown as the most unstable region within the yeast genome (Kobayashi, 2006). Being housed in the nucleolus, double-strand breaks (DSBs) will activate special DNA repair machinery, reduce rRNA transcription, and induce nucleolar cap formation (Korsholm et al., 2019). Although these steps can be successful, recombination events often lead to copy number reductions or mutations. As previously stated in this chapter, genomic instability is linked to organismal aging, and ribosomal DNA repeats are the most susceptible to instability. Hence, the aging hallmarks of protein homeostasis and genome instability are inherently linked.

Of course, the loss of proteostasis is linked to protein synthesis as well, and aberrations in this process can increase in frequency over time (Taylor and Dillin, 2011). Protein synthesis must occur slowly enough for secondary structures to form. Without these, the proper tertiary and quaternary structures could be disallowed. Additionally, the number and type of proteins synthesized over the lifetime has been linked to aging (Selman et al., 2009), partially through results obtained from intermittent fasting which activates autophagy (Chung and Chung, 2019) and has been shown to improve proteostasis (Matai et al., 2019). If initial folding occurs correctly, and the ribosomes are operating at optimal levels, then chaperones and co-chaperones typically assist in forming the tertiary and quaternary protein structures. Co-chaperones do not directly interact with the protein folding itself but assist the chaperones in guiding proteins to their folding sites. Co-chaperones, like heat shock proteins, help chaperones during times of stress, such as anaerobic conditions, acidic conditions, and heat. It has been shown that age-related declines in co-chaperone effectiveness can lead to endoplasmic reticulum stress and chondrocyte apoptosis (Tan et al., 2020).

In addition to protein translation and folding, protein degradation processes become less efficient with age (Vernace et al., 2007). There are several aspects of protein degradation, including lysosomal, proteasomal, and autophagic, with the latter also able to degrade whole organelles (Zhao et al., 2022). Lysosomes are typically highly acidic, at a pH of 4.5, and contain digestive enzymes that optimally function at this pH (Cooper, 2000). During homeostatic processes, both aspects of acid and enzymes are used for protein/organelle degradation, surface receptor recycling, and pathogen sequestration. Ubiquitination is an additional process that targets proteins for degradation through chaperone machinery. Regardless of the molecules being degraded, the internal lysosomal contents require strict regulation to prevent spillage into the cytosol. For reasons still unknown, lysosomal permeability increases in frequency with age (Guerrero-Navarro et al., 2022).

Additionally, the field of autophagy has strong links to aging (Barbosa et al., 2019). Specifically, the ability of cells to undergo accurate and effective degradation of organelles and proteins via autophagic mechanisms declines with age. Coupled with lysosomal permeability, autophagy dysfunction can lead to the accumulation of misfolded proteins and defunct organelles. In fact, during the natural course of aging, any number of these proteostatic processes can breakdown or become less efficient, resulting in a multitude of aging-related phenotypes, including mitochondrial dysfunction and senescent cell accumulation (see below).

Regarding environmental exposures, air pollution has been shown to promote cellular oxidative stress (Lodovici and Bigagli, 2011; Gangwar et al., 2020), protein misfolding (Devi et al., 2021; 2022; Jankowska-Kieltyka et al., 2021), post-translational protein modifications (Watterson et al., 2009; Lodovici and Bigagli, 2011), and aberrant protein degradation (lysosomal (Watts et al., 2022) and autophagy (Park et al., 2018)). Many papers also point to air pollution-associated post-transcriptional modifications to RNAs (Rider and Carlsten, 2019; Gonzalez-Rivera et al., 2020), which would affect downstream protein translation. These result in any number of disease modalities, but particular attention is paid to lung fibrosis (Majewski and Piotrowski, 2020), cardiovascular disease (Gangwar et al., 2020), and ADRD (Kikis, 2020; Calderón-Garcidueñas et al., 2021).

Given that air pollution is inhaled, the initial site of oxidative stress is typically the airway and lungs. As such, the resident populations of macrophages and neutrophils are first-responders to this insult (Becker et al., 2005; Valderrama et al., 2022) and sound the alarm for a more systemic and adaptive immune response (Miyata and van Eeden, 2011). Regardless, endogenous ROS production will increase (Rao et al., 2018) which can affect proteins, and can cause neutrophils to release their cellular contents into the extracellular space—a dramatic event whose end result has been termed a neutrophil extracellular trap (NET). These NETs are known to cause additional oxidative stress and protein damage in the surrounding cells (Beavers and Skaar, 2016). As protein folding mechanisms rely on redox homeostasis (Chong et al., 2017), an abundance of ROS can cause misfolded proteins. In turn, this can cause additional ER stress and activate the unfolded protein response, resulting in additional cellular stress (Grootjans et al., 2016; Plaisance et al., 2016; Chong et al., 2017; Bhattarai et al., 2021). Additionally, protein misfolding can be recognized and sequestered for refolding by chaperones (Hervás and Oroz, 2020) or transferred to protein degradation machinery with the help of co-chaperones (Sontag et al., 2017). After exposure to air pollution, proteosomal activity is decreased (Kipen et al., 2011; Devi et al., 2021) leaving the chaperone and co-chaperone with nowhere to shuttle the misfolded protein. In some cases, the lysosomal and autophagic routes of misfolded protein degradation could be alternatively activated, and have been shown to be increased after air pollution exposure on skin fibroblasts (Park et al., 2018). Moreover, the mechanistic alterations to protein homeostasis after air pollution are coming into focus. However, the individual organ responses (*e.g*., heart, lung, gut, brain, etc.) could differ and their thorough investigation will eventually be required for a complete comprehension of the role of air pollution in protein homeostasis.

## 3 Antagonistic hallmarks of aging–mitochondrial dysfunction, deregulated nutrient sensing, and cellular senescence

### 3.1 Mitochondrial dysfunction

Although the “Loss of Protein Homeostasis” category does include protein degradation, the lysosomal and proteasomal machinery are linked with damage response so intimately that the aging field has designated an entire category to the mitochondrial-lysosomal axis theory of aging (Brunk and Terman, 2002). This axis of aging revolves around the idea that long-lived post-mitotic cells often exist without a stock of stem cells for replenishment. Moreover, these cells will be the most susceptible to age-related phenomena. Cell types in this category include cardiomyocytes and neurons, but any cell that cannot be replaced easily will fall under this classification. To put it lightly, these cells have problems: 1) with age, they cannot degrade protein adducts and organelles; 2) this results in an accumulation of ROS via mitochondrial production; and then 3) these cells cannot be replaced since reinforcements do not exist.

One outcome from these problems is the unintentional formation of lipofuscin through the oxidization of unsaturated fatty acids from mitochondrial ROS production, which have been called “age pigment” and are linked to aging in neurons (Gray and Woulfe, 2005), and cardiomyocytes (Terman and Brunk, 1998). Broadly, lipofuscins are yellow-brown granules that contain biproducts of lysosomal degradation. Moreover, lipofuscin granules appear to be comprised of oxidized proteins and lipids (Double et al., 2008) but can also contain sugar and metals like mercury, aluminum, iron, copper, and/or zinc. Strangely, a comprehensive understanding of lipofuscin composition, distribution, and formation is still sorely lacking. Within the brain, it appears that a decrease in the iron-storing ferritin protein can cause lipofuscin production via the inhibition of the MCOLN1 metal transport channel (König et al., 2017; Boudewyn and Walkley, 2019; Stepien et al., 2020). Although this could be caused by dysfunctions in MCOLN1 rather than a decrease in ferritin production, the end result is the same. Namely, free ferrous ions can interact with lipids and proteins within lysosomes transforming them into lipofuscin. In turn, this impairs the ability of affected lysosomes to perform normal functions and can result in multi-lysosome fusion (Milward et al., 2012).

Indeed, there are many known mitochondrial dysfunctions known to be associated with particulate matter exposure, and some speculate that the olfactory system is the causeway to deteriorating brain health (Chew et al., 2020b). Specifically, decreased mitochondrial oxygen consumption rate (Breton et al., 2019; Chew et al., 2020b), increased mtDNA oxidization (Byun et al., 2016; Grevendonk et al., 2016), altered methylation patterns (Byun et al., 2013; Breton et al., 2019), outer membrane permeabilization (Chew et al., 2020a), and fusion/fission events (Li et al., 2015). Although the large breadth of mitochondrial studies utilized *in vitro* methodologies, the persistence of *in vivo* results reveals a diverse landscape of health effects that should be heeded. A recent review (Reddam et al., 2022) attempted to subdivide the literature from 2012–2022 into chemical exposures of polycyclic aromatic hydrocarbons, air pollutants, heavy metals, endocrine-disrupting compounds, pesticides, and nanomaterials. They applied these classifications to population studies and revealed consistent mitochondrial dysfunction biomarkers, including mtDNA copy number, oxidative damage, outer membrane potential, calcium levels, and ATP levels.

### 3.2 Deregulated nutrient sensing

There are four key pathways affecting aging-associated nutrient sensing, with their component proteins of insulin-like growth factor 1 (IGF-1), mammalian/mechanistic target of rapamycin (mTOR), sirtuins, and AMP (adenosine monophosphate) kinase (AMPK) (López-Otín et al., 2013). Together, insulin and IGF-1 make up the insulin and insulin-like growth factor (IIS) pathway. Attenuation of this pathway appears to increase lifespan in mice, worms, and flies, which implicates the caloric restriction model of aging (López-Otín et al., 2013). When overactive, the IIS pathway can increase cancer risk, metabolic load, and cellular proliferation (Milman et al., 2016). As such, when we reduce IIS early in life, it promotes longevity. However, it appears that the body naturally reduces this pathway during aging and diminishes IIS to the point of detriment once we reach a geriatric state (Milman et al., 2016). Metformin is a drug developed for diabetic patients, which can increase insulin sensitivity (decreases blood glucose levels) and decreases glucose liver production and absorption. As a putative antiaging drug, it was discovered to reduce mitochondrial oxidative stress (partially through sirtuin 3), which increased lifespans in model organisms. Unfortunately, long-term use can lead to vitamin B12 deficiency, liver problems, lactic acidosis, and has been linked to neuropathy (Metformin, 2012; Aroda et al., 2016). The search for a silver bullet continues.

**FIGURE 4:**
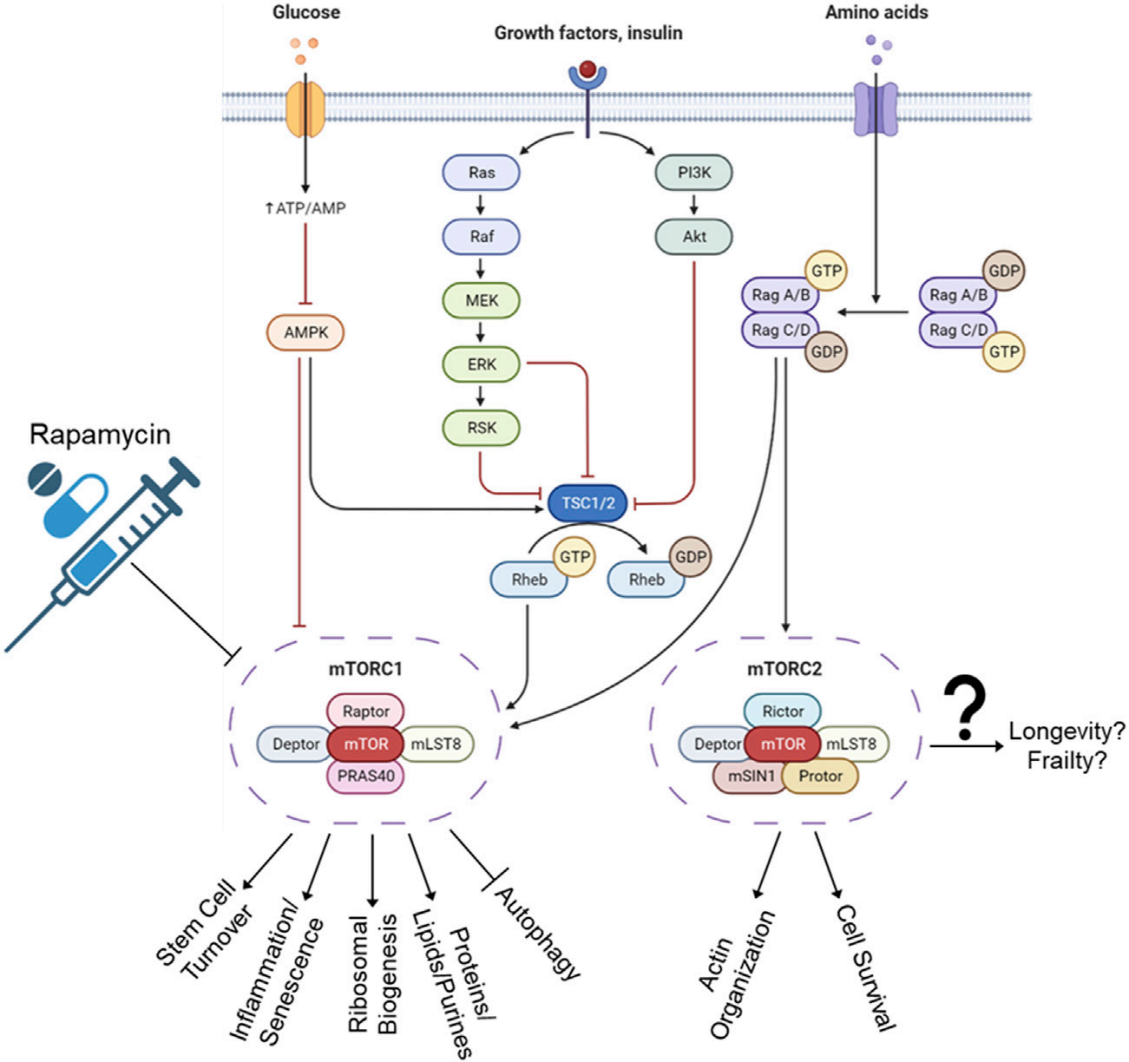
mTORC1 and mTORC2 pathways. The C1 complex regulates cellular processes such as growth, division, and protein synthesis. This activation has been linked to senescent cell accumulation and a decrease in lifespan. Autophagy, a process of energy source selection, is also impacted by C1 activation. The effects of C2 complex are less clear and still debated in the scientific community, with some studies showing it promoting lifespan and others reducing it. The outcomes of C2 can also vary depending on its regulation of factors such as FOXO3a and mTORC2. Rapamycin was developed for immunosuppressive properties and was found to extend lifespan through blocking of mTORC1 complex. Unfortunately, long-term use also inactivates mTORC2, increases blood pressure, elevates cholesterol, and increases risk of blood clots, stroke, and pulmonary embolisms.

The mTORC1 and mTORC2 protein complexes result in different outcomes (Figure 4). The mTORC1 complex responds to amino acids and growth factors by signaling the cell to grow, divide, synthesize proteins, and other inflammation-inducing cellular processes. These processes have been linked to senescent cell accumulation based on cellular division leading to a Hayflick limit, stem cell exhaustion, and accumulation of errors during DNA replication. The mTORC1 complex activation has also been shown to halt autophagy, which is likely an on/off gate that is used to determine which energy source best suits the cellular needs (Rabanal-Ruiz et al., 2017). Namely, if there are nutrients in the environment, then we should not cannibalize excess organelles and lipids via autophagy as an energy source.

The current understanding of mTORC2 complex outcomes will be a shorter list than mTORC1, and whether mTORC2 promotes or reduces lifespan is still debated. Some studies say that mTORC2 also blocks autophagy (Ballesteros-Álvarez and Andersen, 2021), which decreases lifespan. Some publications say it blocks FOXO3a, thereby decreasing proliferation and increasing lifespan (Johnson et al., 2013). While other studies have shown that FOXO3a downregulation leads to age-associated pathologies (Akasaki et al., 2014). Confounding still, murine hypothalamic mTORC2 was shown to increase with age, which prevented obesity, frailty, inactivity, and age (Ballesteros-Álvarez and Andersen, 2021).

Regarding drug interventions, rapamycin was originally discovered on Rapa Nui (Easter Island), and developed initially as an antifungal (Vézina et al., 1975). Later, it was developed for its immunosuppressive—and hence, organ rejection suppression—abilities (Baroja-Mazo et al., 2016). It was later discovered that rapamycin extended lifespans in model organisms, and humans jumped at the opportunity to fill their bodies with the unknown. Unfortunately, long-term rapamycin will also inactivate mTORC2 (Sarbassov et al., 2006). It also *could* increase arterial pressure, elevate cholesterol and/or other lipids (Sacks, 1999; Kumar et al., 2019), and *does* increase risk of thrombosis, venous thromboembolism, and clotting problems (Camici et al., 2010; Ahya et al., 2011). The search for a silver bullet continues.

Sirtuins are a particularly fascinating category of enzymes, which also have a deep connection with aging. Originally discovered in yeast, these are currently classified as Class III histone deacetylases (HDACs): a group of histone deacetylases that have the ability to remove acetyl groups from histones, proteins, and transcription factors in an NAD^+^-dependent manner (Braunstein et al., 1993). However, sirtuin functions have been expanded to NAD^+^-dependent mono-ADP ribosyltransferases, lipoamidase, desuccinylase, demalonylase, and deacylase activity (Chang et al., 2020). The HDAC classification comes under further fire based on publications showing that sirtuins can remove moieties other than acetyl groups, and that their catalytic activity increases proportionally to the size of aliphatic tail being removed (Gertler and Cohen, 2013). Meaning, some sirtuins will have a preference for butyryl, palmitoyl, and myristoyl over acetyl groups. As such, Jiang et al. (2013), are proposing to rename the sirtuins to deacylases. Arguably, this recategorization would be misleading, as not all sirtuins have deacylase ability. To authentically recategorize sirtuins, it would be more reasonable to categorize sirtuins based on cellular compartment localization, or we could recategorize sirtuins by their individual functions. Regardless, each sirtuin will be partially explained below and their known functions are summarized in Chang et al. (2020).

Unfortunately, the expression levels and effect sizes of sirtuins varies wildly in our current model organisms (López-Otín et al., 2013), so we will be forced to focus on the most important animal. Humans have 7 sirtuins (named SIRT1-SIRT7). In young and middle-aged humans, low cellular energy reserves cause an increase in NAD^+^ levels, a mechanism which is impaired in older adults (McReynolds et al., 2020). Sirtuins sense this increase, and some can control catabolic metabolism. More specifically, SIRTs 1 and 2 can translocate between cytoplasm and nucleus; SIRTs 6 and 7 are said to exclusively reside in the nucleus; and SIRTs 3, 4, and 5 localize to the mitochondria (Liszt et al., 2005; Ahuja et al., 2007; Buler et al., 2016). Notably, SIRT2 is somewhat of a nomadic enzyme, able to translocate between the cytoplasm, nucleus, and mitochondria, but is most often found in the cytoplasmic compartment (Liu et al., 2017). All SIRTs have deacetylase abilities and others, but SIRT 4 and 6 are the ADP-ribosyltransferases (Liszt et al., 2005; Ahuja et al., 2007; Buler et al., 2016).

SIRT1 is an ortholog of the gene for SIRT2, and is involved in neural development through axonal elongation, neurite growth, and dendrite branching; synaptic plasticity for memory formation; hypothalamic functions affecting feeding behaviors, endocrine functions, and circadian rhythm; protects against Alzheimer’s, Parkinson’s, and motor neuron disease; and confers stress resistance, metabolic homeostasis, and genomic stability (Herskovits and Guarente, 2014). SIRT1 has secondary activation effects, including activation of PARP for DNA repair, TFAM for mitochondrial biogenesis, FOXO3a for antioxidant effects, and NRF2 which blocks NFκB and activates SOD for downstream secondary activation of antioxidant genes.

With the nomadic behavior of SIRT2, the ability to perform intercompartmental tasks presents itself. Specifically, SIRT2 is associated with senescence (Anwar et al., 2016), cytoskeletal stabilization (North et al., 2003; Harting and Knöll, 2010; Maxwell et al., 2011; Silva et al., 2017; Esteves et al., 2019), myelin formation (Beirowski et al., 2011), oligodendrocyte differentiation (Tang and Chua, 2008), autophagy (Gal et al., 2012; Inoue et al., 2014; Liu et al., 2017; Silva et al., 2017; Esteves et al., 2019), inflammation (Mendes et al., 2017), upregulation during G_2_/M mitotic transition (Sola-Sevilla et al., 2021), and is required for proper cytokinesis (Tang and Chua, 2008). Among the sirtuins, SIRT2 has the strongest neurological presence, with expression seen in neurons, oligodendrocytes, astrocytes, and microglia (Jayasena et al., 2016). Based on the neurological potential, autophagy and senescence implications, and wide localization for a multitude of histone deacetylations, SIRT2 has been proposed as central to human aging. Unfortunately, the increase/decrease in SIRT2 abundance within different brain compartments is riddled with conflicting studies (Sola-Sevilla et al., 2021). Namely, three splice variants have been discovered, but mRNA and proteins levels are seen to increase, decrease, or stay the same with age (Maxwell et al., 2011; Kireev et al., 2013; Braidy et al., 2015; Garg et al., 2017; Diaz-Perdigon et al., 2020). When examining the breadth of data available, a clearer picture can be resolved after removing conflicting data in rats and mice. In humans, the studies show an age-related increase in plasma SIRT2 mRNA (Yudoh et al., 2015), with a decrease in peripheral blood mononuclear cell (PBMC) protein abundance (Wongchitrat et al., 2019). Unfortunately, the neurological implications remain unclear, and this does not speak to regional abundances.

Pharmacologically, few studies have upregulated SIRT1 or SIRT2 after air pollution exposure, but some have performed correlation studies with SIRTs and air pollution. A longitudinal study from China saw a significant correlation with hazard ratios from particulate matter exposure and levels of SIRT1 polymorphisms (Yao et al., 2021). Another study revealed pre-conception air pollution exposure increased SIRT1 and SIRT2 mRNA in offspring after birth. These data indicate a potential linkage in anti-oxidant effects of these sirtuins, or the epigenetic modification capabilities (Tanwar et al., 2018).

SIRT3 is one of the 3 mitochondrial sirtuins and has been implicated in neurological and cardiovascular diseases (Silaghi et al., 2021) as well as having protective effects after urban air pollution exposure (Chen et al., 2016). These findings are largely based on the age-related decline in activity leading to increased susceptibility of endothelial dysfunction, hypertension, and heart failure. Furthermore, proteomic data shows that it regulates 100s of proteins (Rardin et al., 2013; Yang et al., 2016; Carrico et al., 2018), involving β-oxidation, antioxidant proteins, glutathione production, metabolism of amino acids, and mitochondrial permeability (Hazelton et al., 2009; Cheng et al., 2016). As the primary mitochondrial deacetylase, it removes the acyl group from most mitochondrial proteins. In fact, it is the largest contributor to the mitochondrial “acetylome”, whereby deletion of SIRT3 (but not 4 or 5) will result in protein hyperacetylation (Lombard et al., 2007). In humans, polymorphisms that upregulate SIRT3 increased longevity (Albani et al., 2014), and increased expression in populations have been shown to extend human lifespan (Rose et al., 2003; Halaschek-Wiener et al., 2009). Based on its antioxidant effects, a clear link to aging can be seen. Through the complexing of SIRT3 with FOXO3a, SOD2 upregulations occur, which reduce ROS (Silaghi et al., 2021). But, if SIRT3 is exclusively found in the mitochondria, and the SOD gene is found in the nucleus, a question can be proposed of whether SIRT3 has a nomadic tendency as well. Independently of these musings, SIRT3 decreases mitochondrial membrane potential and ROS production while increasing cellular respiration. Conversely, downregulation of SIRT3 was shown to induce TGF-β synthesis, resulting in activation of NFATc and beta-catenin (pro-fibrosis). This mechanism is largely known, whereby SIRT3 indirectly downregulates TGF-β by inhibiting the transcription factor c-Jun (Sundaresan et al., 2015). Within the heart, TGF-β differentiates cardiac fibroblasts to myofibroblasts for the deposition of extracellular matrix, and the formation of fibrotic tissue. Indeed, patients with end-stage heart failure showed a significant decrease in SIRT3, resulting in an increase of TGF-β production (Sundaresan et al., 2015). Furthermore, hyperlipidemia has been shown to induce macrophage-based atherosclerosis. These monocytes downregulate SIRT3, leading to overabundance of acetylated ATG5 which decreases autophagic processes. In turn, this increases NLRP3 inflammasome production, and IL-1β secretion for plaque formation. Taken together, SIRT3 is an exciting target for therapeutic intervention, but the sheer number of outcomes associated with SIRT3 requires careful investigation.

Although we know that SIRT4 is localized within the mitochondria, its activities are still somewhat mysterious (Min et al., 2019). This can partially be explained by the tissue- and cell-specific activities already observed. For example, pancreatic SIRT4 has been shown to inhibit insulin secretion. Within the muscles, adipose, and liver, it suppresses fatty acid oxidization. However, the oxidization mechanism appears to be different from tissue to tissue as well. In muscles and fat, SIRT4 appears to block the malonyl-CoA decarboxylase, resulting in fatty acid synthesis rather than oxidation (Nasrin et al., 2010; Acs et al., 2014; Zeng et al., 2018). In the liver, SIRT4 has been shown to block the interaction between SIRT1 and PPARα, leading to a decrease in fatty acid oxidization (Nasrin et al., 2010). Unfortunately, these studies were performed in mice, which disallows human pathway translations or conclusions.

The first review of SIRT5 came out in 2016 (Yang et al., 2017) after a string of discoveries showed weak deacetylase activity, but strong desuccinylase, demalonylase and deglutarylase activities (Du et al., 2011), among others. After preliminary links were formed between SIRT5 abundance and incidence of Alzheimer’s, cancer, and Parkinson’s disease, the door of excitement blew open for a rash of new peptide and small-molecule inhibitors. Unlike every other SIRT, SIRT5 activation and overexpression was initially believed to be deleterious. Studies showed that overexpression led to accelerated aging and damage, rather than increasing longevity. For example, SIRT5 was amplified by 30% in ovarian carcinoma (Bell et al., 2011), was overexpressed in non-small cell lung cancer (Lu et al., 2014), and protein levels were shown to increase in tandem with Alzheimer’s progression (Lutz et al., 2014). Furthermore, a SNP in SIRT5 increased protein abundance, and appeared to contribute to mitochondrial dysfunction. The authors related these changes to Parkinson’s and other age-associated disease states through gene activation profiles (Glorioso et al., 2011). However, more recent studies have found counter evidence showing that SIRT5 depletion causes mitochondrial dysfunction, expression does not increase with Alzheimer’s progression, and can only be linked to Parkinson’s via an autophagic degradation of SIRT5 itself (Baeken et al., 2021; Haschler et al., 2021; Wu et al., 2021). Arguably, a beneficial effect of SIRT5 seems more likely based on the positive effects of SIRTs 1, 2, 3, 4, 6, and 7.

But rather than attempting to fully understand the functionality of SIRT5, the field is sprinting ahead with inhibition strategies in mice through small molecules and peptides. As of 2017, there were 6 peptide inhibitors, and 7 small molecule inhibitors. Through genetic engineering, a SIRT5 knockout group showed an increase in lifespan and decrease in tumor incidence within a mouse breast cancer model (Abril et al., 2021). However, these findings of SIRT5 knockouts and deleterious outcomes should be taken with a huge grain of salt until studies are repeated and mechanisms of action are elucidated.

Unlike SIRT5, the field believes SIRT6 to have antitumor properties through its roles in regulating gene expression, metabolism, and DNA repair. However, these effects can be lost after air pollution exposure, evidenced by an *in vivo* inhibition of SIRT6 mRNA in the lungs (Ribeiro et al., 2017) As stated earlier, SIRT 6 is proposed as a permanent resident of the nucleus. One outcome is the potential to directly impact DNA repair. This is achieved through ADP-ribosylation of K521 residue on PARP1, which engages the dsDNA break repair (Mao et al., 2011). Further, SIRT6 has a high binding affinity for NAD^+^, which allowed a systematic evaluation of binding partners. Results from these experiments include the deacetylation of H3K9 and H3K56 in gene promoter sites (Kugel and Mostoslavsky, 2014), co-repression of nuclear factor κB (NFκB) and hypoxia-inducible factor 1α (HIF-1α) (Kawahara et al., 2009; Zhong et al., 2010), telomere maintenance (Michishita et al., 2008), and co-repression of transcription factor c-MYC (Sebastián et al., 2012), among many others. Interestingly, the diacylation activity of SIRT6 is wide-ranging as well, and includes modifying the inflammatory master regulator TNF-α (Jiang et al., 2013). More specifically, the secretion of TNF-α requires SIRT6 to remove the fatty acyl groups from K19 and K20 (Jiang et al., 2013). Without this diacylation, TNF-α is relegated to the lysosomes for degradation. More recently, SIRT6 has been shown to deacylate R-Ras2, which prevents the complexing to PI3K and thereby inhibits proliferation (Zhang et al., 2017). Thus, SIRT6 has a plethora of therapeutic potential, but this widespread activity also increases the potential for off-target effects through pharmacological modulation (Chang et al., 2020). As an opportunity for researchers investigating mitotic bookmarks, lower levels of SIRT6 correlate with mitotic defects (Tasselli et al., 2016), indicating the potential for SIRT6 as a yet-uninvestigated mitotic bookmark.

SIRT7 is known to localize within the nucleus and nucleolus and interacts with RNA polymerase I (Lombard et al., 2007). It is currently believed that SIRT7 has a role in DNA repair, whereby it accumulates to the site of dsDNA breaks and deacetylates H3K18ac (Barber et al., 2012; Vazquez et al., 2016; Zhang et al., 2016). This results in the binding of double-strand break repair protein-1 for nonhomologous end joining. Additionally, SIRT7 has been implicated in cardiomyocyte stress resistance (Vakhrusheva et al., 2008), and reductions in liver endoplasmic reticulum stress through suppression of Myc activity (Shin et al., 2013).

Since these molecules are NAD-dependent, a small review of NAD^+^ should be endeavored. NAD^+^ is generated *in vivo* through the *de novo* pathway from amino acids, or the salvage pathway from nicotinamide. At last count on Pathbank, there are 6 main branching pathways that lead back to mitochondrial production of NAD^+^ via nicotinamide mononucleotide adenylyltransferase1 (NMNAT3), with further production available in the nucleus, and Golgi via NMNAT1 and NMNAT1, respectively. Increasing these branches to a point of convolution, hundreds of compounds are known to feed into the *de novo* and salvage pathways. From convolution to simplicity, we know that the *de novo* pathway is regulated by a rate-limiting enzyme, nicotinamide phosphoribosyltransferase (NAMPT). The NAMPT enzyme has its own circadian regulator complex consisting of a circadian locomotor output cycles kaput (CLOCK) and a brain and muscle aryl hydrocarbon receptor nuclear translocator-like 1 (BMAL1) (Nakagawa and Guarente, 2011; Grabowska et al., 2017). Without the proper abundance of feeder molecules, circadian time of day, and amount of rate-limiting enzyme, NAD^+^ metabolism can be skewed. In turn, this could cause an unintended decrease in sirtuin activity.

With regard to air pollution, these essential factors may be altered, but few studies have examined the link between sirtuins two to seven with air pollution/particulate matter. As of writing, the following results were found from a PubMed query for “(‘sirtuin [x]’ OR ‘SIRT [x]’ OR ‘sirtuin-[x]’) and (‘air pollution’ OR ‘particulate matter’)” where x represents the numerical sirtuin in question. SIRT1: 28 publications; SIRT2: 4; SIRT3: 5; SIRT4: 0; SIRT5: 0; SIRT6: 1; SIRT7: 0.

Of note, a longitudinal human cohort study (*n* = 7,083 participants) correlated air pollution levels with all-cause mortality and revealed a significantly higher hazard ration in women carrying two SIRT1_391 alleles (Yao et al., 2021). In mice, air pollution exposure *in utero* caused reduced cardiac expression levels of SIRT1 and SIRT2, with physiological outcomes of adult heart failure, remodeling, and epigenetic changes. SIRT3 also appears to have a cardioprotective effect in mice based on a SIRT3 KO model compared to WT controls (Song et al., 2021), and melatonin supplementation could offset some of the cardiac fibrosis effects from PM_2.5_ by regulation SIR3-mediated SOD2 deacetylaton (Jiang et al., 2021). For SIRT6, young (2 months) and old animals (15 months) were exposed to diesel exhaust for 30 days. In young animals, lung SIRT6 gene expression was decreased, while protein expression increased. No effects in SIRT6 were observed in old animals (Ribeiro Júnior et al., 2019).

Finally, AMP kinase is thought to extend lifespan through regulation of metabolic homeostasis and stress resistance (Salminen and Kaarniranta, 2012). Studies have shown increased in AMPK activity stimulate the signaling pathways of FoxO, Nrf2, and SIRT1, as well as downregulation of NF-κB, which reduces inflammatory responses. Furthermore, this responsiveness and activity declines with age, allowing for chronic, unresolved, low-grade inflammation. In turn, this inflammation has been linked to senescence, metabolic disorders, and age-related diseases like cancer and Alzheimer’s.

With regard to environmental stressors, heavy metal exposure from cadmium, lead, and mercury (among others) have been implicated with metabolic syndrome (Planchart et al., 2018), possibly through an increased insulin resistance (Su et al., 2021) and islet cell damage (Chen et al., 2009). Metabolic syndrome is characterized as having a minimum of 3/5 of the following: abdominal obesity, high blood pressure, impaired fasting glucose, high triglyceride levels, and low HDL cholesterol levels. Overall, metabolic syndrome has been partially linked to accelerated cellular aging (Kong et al., 2013), but more evidence is necessary before solid conclusions to be made.

### 3.3 Cellular senescence

Senescence is an irreversible state of proinflammatory cell cycle arrest. During natural aging, mammals accumulate senescent cells. Upon senescing, cells alter their secretome (Coppé et al., 2008), which has been characterized as the senescence-associated secretory phenotype (SASP). These factors (like IL-6, IL-8, and TNFα) can cause paracrine senescence (Borghesan et al., 2019), which can feed forward senescent cell accumulation (Teo et al., 2019). Studies have shown SASPs to be cell-type specific (Hudgins et al., 2018; Basisty et al., 2020), species-dependent (Hudgins et al., 2018), stimulus-specific (Martínez-Zamudio et al., 2017), and age-dependent (Basisty et al., 2020), but broadly link senescence with age-associated pathologies.

Environmental stressors can promote neurological senescence, but the mechanisms underlying this relationship are unclear (Chinta et al., 2018) and the ability of post-mitotic neurons to undergo senescence is still debated (Chinta et al., 2013). However, neuronal progenitors can undergo senescence (Rachmian and Krizhanovsky, 2023), as well as oligodendrocyte progenitors (Ogrodnik et al., 2021), astrocytes (Simmnacher et al., 2020), microglia (Hu et al., 2022), and neuro-associated endothelial cells and pericytes (Yamazaki et al., 2016). Additionally, the herbicide paraquat is associated with idiopathic Parkinson’s disease, potentially through a skewing of neurological astrocytes towards the senescent phenotype (Chinta et al., 2013; Chinta et al., 2018; Scott and Williams-Gray, 2018). Within other tissues, air pollution has been shown to increase senescence in the lung (Mora and Rojas, 2013; Chang-Chien et al., 2021; Eckhardt and Wu, 2021). However, any organ susceptible to the immediate and downstream effects of particulate matter insult (Schraufnagel et al., 2019) will also have an increased risk for senescence, including eyes (Sreekumar et al., 2020), heart (Chen et al., 2022), stomach (Penfield et al., 2013), bone (Pignolo et al., 2021), mouth and jaw (Parkinson and Prime, 2022), and reproductive health (Lemaître and Gaillard, 2017). These senescent cells can contribute to pathology, meaning inhaled toxicants may indirectly or directly augment those outcomes.

## 4 Integrative hallmarks of aging–intercellular communication and stem cell exhaustion

### 4.1 Intercellular communication

Cellular communication and hormone balance alters as we age. This results in a skewing towards a more inflammatory state, resulting in osteoporosis, sarcopenia, cognitive decline, stem cell exhaustion (see below), and others (López-Otín et al., 2013). This hallmark has been modulated through dietary caloric restriction (Lee et al., 2006; Pifferi et al., 2018), parabiosis (connecting the circulation of old mice to young), and apheresis (removal of blood), but the human lifespan effects are difficult to determine (Mattison et al., 2017). Mechanistically, the increase in NFκB within the hypothalamus causes a reduction in gonadotropin-releasing hormone (GnRH), leading to some of the aforementioned effects. Additionally, SASP factors have been implicated in this breakdown of altered communication through their consistent recruitment of immune cells, lack of clearance, and proinflammatory state (Palmer et al., 2015; Childs et al., 2016; Xu et al., 2017).

Regarding environmental toxicants, a longstanding body of research has linked exposures with altered intercellular communication (Peters et al., 2021). The alterations are typically thought of in the context of SASP alterations through exposures like ozone (Feng et al., 2016), and alterations to extracellular vesicle payloads through exposures like air pollution (Bollati et al., 2015). However, environmental toxicant exposure can alter the ligandome (Tian et al., 2020), and chemical mixtures are being employed to determine receptor changes that would alter endocrine signaling (Vinggaard et al., 2021). Summarily, EVs and SASP release are factors that should be brought into context with the receptor/ligand alterations that occur from multiple environmental toxicants.

### 4.2 Stem cell exhaustion

Stem cell exhaustion is directly responsible for age-related problems of frailty and a weakened immune system. However, there are multiple causes of this phenotypic outcome. To this point, we have observed aging phenotypes arising from accumulation of ROS, toxic metabolites, DNA damage, epigenetic alterations, damaged proteins, and mitochondrial dysfunction. All of these can contribute to stem cell exhaustion in addition to many of the other hallmarks listed in Figure 1. For example, the pro-inflammatory cytokines released from senescent cells will suppress the proliferation of stem cells, which contributes to the age-related reductions in tissue regeneration and cellular turnover. Another aspect of exhaustion occurs from telomere shortening (Hiyama and Hiyama, 2007) which can result in a replicative senescence within the stem cell population. The existence of senescent cells within signaling distance of other stem cells will promote further reductions in stem cell activity through SASP factor release. Additionally, both white and red blood cell production occurs through the proliferative ability of stem cells. Neutrophils are short-lived and are known to return to the bone marrow niche after several hours in circulation. They are welcomed home by macrophages who cannibalize them. This triggers macrophages to express a liver x receptor (LXR), which blocks CXCL12 production in the hematopoietic bone marrow niche. Through this blockage, stem cells begin differentiation and are released as hematopoietic progenitor cells in the blood (Casanova-Acebes et al., 2013).

Over the natural course of aging, the abundance of immune cells declines. This reduction in available immune cells decreases the body’s ability to fight off pathogenic insults as we age, leading to multiple diseases. Although science has been unable to reverse this effect, many researchers are examining the blood and its contents. Seminal work from the R.A. Lambert in 1911 revealed animals could have their circulatory systems surgically conjoined (parabiosis) (Lambert, 1911). For over a half-century, parabiosis studies were performed based on age-matched exposure and control animals (Binhammer et al., 1953; Warren et al., 1960; Carroll and Kimeldorf, 1967; Carroll and Kimeldorf, 1969). In 1957, the circulatory systems of young and old mice were surgically integrated (heterochronic parabiosis), and an observable lifespan increase was achieved (Mccay et al., 1957; Hrůza, 1971; Ludwig and Elashoff, 1972). Frankensteinian parabiosis has been deemed immoral and unethical in humans. As such, we must examine the blood factors themselves and attempt intervention. Pioneering work by the Wyss-Coray lab illustrated beneficial neurological effects from young plasma transfusions in old mice (Villeda et al., 2014). Similarly, researchers injected a blood plasma cocktail meant to mimic the effects of parabiosis, and reverted the epigenetic age of liver by 73.4%, blood and heart by 52%, and hypothalamus by 11% (Yousef et al., 2015), and plasma dilution (rather than transfusion) has been shown to be effective in mice (Mehdipour et al., 2020; Mehdipour et al., 2021). Not wanting to wait for government approval, a group of Russian biohackers took it upon themselves to undergo the first human tests. Their results showed an improvement in liver markers, increase in naïve T-cells, and the myelocyte/lymphocyte ratios improved. Unfortunately, the sample size of 2 called these results into question until a clinical trial performed plasma dilution and achieved similar results (Kim et al., 2022). Namely, reductions in DNA damage marker 8-OHdG and cellular senescence marker p16 in PBMCs, and a rejuvenation of the myelocyte/lymphocyte ratio. Finally, proteomic comparative analyses revealed the potential aging biomarkers of uPAR, TRAIL R1, IL-16, TIMP-1, IL-15R alpha, CD27, APJ, TNFRSF27, CCL25, and TGFBR2.

Regarding air pollution, blood is the highway between organ systems, and is the current best guess as to why non-outward-facing organs are affected after exposure. Ongoing research is attempting to determine whether inhaled smoke particles themselves are entering the blood stream, or if fragmented peptides from lung responses are causing peripheral organ effects. Later this year, Andrew Ottens at Virginia Commonwealth will be publishing proteomics results from mouse hippocampi after wildfire exposure. He was unable to directly determine whether the wildfire particles were reaching the brain. However, pathway analysis revealed neurological damage and aggrephagy–a process of selective autophagic degradation of protein aggregates. Although these results do not preclude particulate matter escaping lungs and entering circulation, this is strong evidence that fragmented peptides are reaching the brain and being cleared.

Another study showed that firefighters who donated blood reduced the circulating levels of “forever chemicals” like perfluoroalkyl and polyfluoroalkyl substances (Gasiorowski et al., 2022). Somewhat gloomily, EPA scientists now say that it is no longer safe to drink rainwater anywhere on the face of this planet due to the presence of these forever chemicals (Cousins et al., 2022). So, the take-away could be to donate more blood, but then the recipient of that blood will receive those chemicals. Broadly, it could be assumed that the biohackers’ results were due to a similar reduction in harmful circulating substances. Regardless, both studies could reduce the amount of stem cell exhaustion by way of removing inflammatory markers that prevent stem cell functions from occurring.

## 5 The interaction between wildfire smoke and the hallmarks of aging

Environmental disasters such as wildfires pose an increasing threat to a growing and aging global population (WHO, 2017). Wildfires produce complex mixtures of inhaled toxicants that can not only damage the lungs but also promote systemic health effects. Short-term pulmonary, cardiovascular and neurological outcomes from inhaled toxicants are well studied, as are some of the implications for chronic diseases such as atherosclerosis. However, relatively little is known about the impact of inhaled toxicants from wildfires on aging—and the further implications for priming age-related disease sequelae, particularly in the brain.

Striking parallels exist between molecular changes within the blood induced by aging and inhaled toxicants. Circulating proteins, such as MMPs, serpins, and inflammatory factors, have been documented to increase with aging and directly promote age-related neurological pathologies (Lehallier et al., 2019; Pluvinage and Wyss-Coray, 2020). Similarly, we have documented that inhaled pollutants (ozone, particulates, complex emissions) cause overlapping compositional and functional alterations to the circulation, and those changes promote vascular and neurological inflammation, which in turn prime age-related pathologies (Channell et al., 2012; Aragon et al., 2017; Mostovenko et al., 2019). Downstream consequences of the circulatory changes following inhalation of particulates and gases include breakdown of the blood-brain barrier (BBB) and activation of microglia and astrocytes (Scieszka et al., 2022). Recent studies of aging note a clear role for vascular cell adhesion molecule-1 (VCAM-1) as a vital intermediate in aging-related BBB breakdown and neurological sequelae (Yousef et al., 2019), and serum obtained after exposure to particulates and gases (in both humans and animals) directly upregulate endothelial VCAM-1 (Channell et al., 2012; Aragon et al., 2016; Tyler et al., 2016). Furthermore, while age-related changes in the circulating proteome provide fundamental revelations, there is another unstudied set of bioactive factors induced within the blood - the peptidomic fraction. We have demonstrated how circulating peptide fragments arise from activated proteases in the lung following inhalation of particulate and gaseous toxicants, retain 2° structure and act through ligand-receptor endothelial interactions to compromise BBB integrity and promote inflammation (Mostovenko et al., 2019). Our laboratory and others have demonstrated that advanced age confers vulnerability to many of the neuroinflammatory and vascular outcomes of inhaled toxicants (Mumaw et al., 2016; Tyler et al., 2018). However, gaps remain in our understanding of the underlying mechanisms of aging and environmental interactions that compromise the neurological and cardiovascular healthspan. Furthermore, how environmental exposures promote aging processes remains understudied, especially in terms of high-concentration insults as seen in wildfire scenarios. Preliminary evidence shows that inhaled wildfire smoke (WFS) accelerate markers of and neurological aging (Scieszka et al., 2022) and reduce learning capability (Cleland et al., 2022). This is likely occurring through the augmentation of circulatory factors that 1) compromise vascular and BBB integrity to induce chronic neuroinflammation and age-associated proteinopathy-related outcomes and 2) induce a reduced metabolic, senescent cellular phenotype that may be treatable with combined anti-inflammatory and NAD + boosting compounds.

## 6 Discussion

The intricate interplay between aging and environmental exposures, as explored in this review, underscores the need for future research on multiple fronts. A critical area of inquiry lies in the further examination of the molecular and cellular mechanisms underpinning the interactions between aging hallmarks and environmental stressors, e.g., inhaled toxicants from wildfires. Although the repercussions on circulatory alterations, neuroinflammation, and BBB integrity have been established and are related to ADRD, specific pollutant effects on cellular/molecular aging pathways involved remain largely elusive. Therefore, uncovering these could lead to targeted interventions and potential therapeutic approaches.

One such prospect is the exploration of specific compounds that could counter the adverse effects induced by wildfire smoke and other inhaled pollutants. In this context, our preliminary findings point towards the potential of combined anti-inflammatory and NAD + boosting compounds. These could act by mitigating inflammation and restoring the reduced metabolic activity in senescent cells induced by wildfire smoke. Therefore, rigorous testing of these compounds in preclinical and clinical settings could help establish their therapeutic efficacy and safety.

Moreover, the intriguing concept of circulating peptide fragments arising from activated proteases in the lung and their subsequent role in promoting inflammation and BBB compromise deserves more in-depth investigation. This could entail studying the peptide fragments’ structure, their interactions with endothelial receptors, and their potential modulation to mitigate their harmful effects.

Beyond mechanistic inquiries, our findings also call for policy action. Given the growing and aging global population and the increasing prevalence of wildfires and other environmental disasters, addressing the health implications of environmental exposures is a public health imperative. Regulatory bodies should consider implementing stringent measures to manage these disasters and reduce public exposure to their harmful consequences. Furthermore, health education campaigns can raise awareness about these health risks and strategies to minimize exposure.

Finally, the role of lifestyle interventions, such as dietary caloric restriction, in modulating the aging hallmarks affected by environmental exposures, warrants further exploration. Long-term, population-level studies could provide valuable insights into their potential benefits.

In conclusion, our review highlights the need for a multidisciplinary approach to tackling the complex problem of environmental exposure-induced acceleration of aging mechanisms. Through concerted efforts spanning basic research, therapeutic development, and policy action, we can aspire to mitigate the detrimental health consequences of such exposures and ensure healthy aging in our society.

## 7 Caveats

While striving to be comprehensive through literature searches, this review is not intended as a systematic review and has likely overlooked some information that could enrich the discussion. In focusing primarily on woodsmoke, we acknowledge that many emerging environmental toxicants warrant their own reviews. This targeted scope allowed a deeper exploration of woodsmoke’s impacts but means the effects of other pollutants require additional attention in future work. Overall, we aimed to advance the conversation on woodsmoke’s effects while recognizing this review’s limitations in breadth. Further research across the spectrum of environmental exposures will continue illuminating their influences on human and ecological health.
